# Alternating-direction method of multipliers estimation of attenuation and activity distributions in time-of-flight flat-panel positron emission tomography

**DOI:** 10.1186/2197-7364-2-S1-A40

**Published:** 2015-05-18

**Authors:** Yueh Hsu, Cheng-Ying Chou

**Affiliations:** National Taiwan University, Taipei, Taiwan

A quantitative reconstruction of radiotracer activity distribution in positron emission tomography (PET) requires correction of attenuation, which was typically estimated through transmission measurements. The advancement in hardware development has prompted the use of time-of-flight (TOF) to improve PET imaging. Recently, the application of TOF-PET has been further extended to obtain attenuation map in addition to activity distribution simultaneously by use of iterative algorithms. Two flat-panel detectors are employed thus many transaxial lines of response are not detected. In this work, we applied the alternating-direction method of multipliers (ADMM) to simultaneously reconstruct TOF-PET and attenuation estimation in a dualhead small-animal PET system. The results were compared with those obtained by use of the maximum-likelihood algorithm. The computer simulation results showed that the application of the ADMM algorithm could greatly improve the image quality and reduce noisy appearance.

